# Small blue round cell tumor of the interosseous membrane bearing a t(2;22)(q34;q12)/*EWS-CREB1 *translocation: a case report

**DOI:** 10.1186/1755-8166-3-12

**Published:** 2010-07-02

**Authors:** Marina Pacheco, Douglas E Horsman, Malcolm M Hayes, Paul W Clarkson, Hassan Huwait, Torsten O Nielsen

**Affiliations:** 1 Department of Pathology and Laboratory Medicine, University of British Columbia, Vancouver, Canada; 2 Department of Pathology, British Columbia Cancer Agency, Vancouver, Canada; 3 Division of Surgical Oncology, British Columbia Cancer Agency, Vancouver, Canada

## Abstract

**Background:**

The group of small blue round cell tumors encompasses a heterogeneous group of neoplasms characterized by primitive appearing round cells with few distinguishing histologic features.

**Results:**

We report the case of a small blue round cell tumor with an *EWS *gene rearrangement detected by fluorescent in situ hybridization (FISH) analysis that mimicked Ewing sarcoma, but with unusual histology and immunohistochemical features. Multi-color karyotyping identified the presence of a t(2;22)(q34;q12) that was initially expected to represent a variant *EWSR1-FEV *translocation. After an extensive workup, the lesion is considered to represent a clear cell sarcoma harboring an *EWSR1-CREB1 *fusion transcript.

**Conclusions:**

This case appears to represent a rare variant of clear cell sarcoma arising in peripheral soft tissues with unusual histology and unique immunophenotype. In this circumstance, FISH for all *EWSR1 *translocation partners or RT- PCR for a spectrum of possible transcript variants is critically important for diagnosis, since cytogenetic analysis or clinical FISH assay using only commercial *EWSR1 *probes will be misleading.

## Background

The accurate diagnosis of small blue round cell neoplasms can be difficult. The differential diagnosis includes sarcomas (such as the Ewing family of tumors (EFT), alveolar rhabdomyosarcoma, poorly differentiated synovial sarcoma, myxoid/round cell liposarcoma, desmoplastic small round cell tumor, and cellular variants of extraskeletal myxoid chondrosarcoma), small cell and lymphoblastic lymphomas, neuroblastoma, melanoma and small cell carcinoma among others.

Although certain histologic features may be useful in differentiating these entities, their general morphology is generic by light microscopy and a large battery of ancillary studies is required. Immunohistochemistry is the first line supplemental methodology and is sufficient for diagnosis in many cases of small round cell tumors. For example, myogenin and myoD1 are specific and sensitive for the diagnosis of rhabdomyosarcoma [1] and lymphoid markers such as CD20, CD3, CD30 and CD45 are very useful in the diagnosis of lymphoma. However, many other markers, although helpful, are not so specific and require interpretation in the context of an immunohistochemical panel. For example, epithelial markers are essential for the diagnosis of carcinoma, but they can also be positive in poorly differentiated synovial sarcoma, Merkel cell carcinoma and in rare Ewing family tumors [2]. S-100 is positive in melanoma but also in clear cell sarcoma, myxoid liposarcoma, extraskeletal myxoid chondrosarcoma and some Ewing family tumors; desmin is strongly positive in rhadomyosarcoma but is also positive in desmoplastic small round cell tumor; and CD99 immunoreactivity is seen in EFT, but also in mesenchymal chondrosarcoma and lymphoblastic lymphoma [3].

FISH analyses for chromosomal translocations can be extremely helpful in this setting, but even these findings may not be specific. *EWSR1 *gene rearrangement is characteristic of EFT, but is also present in extraskeletal myxoid chondrosarcoma, desmoplastic small round cell tumor, and a subset of myxoid/round cell liposarcomas [4]. *FUS *rearrangements are seen in a majority of myxoid/round cell liposarcomas but also in EFT [5,6].

Karyotype analysis is a global genome scan that has the ability to detect gross chromosomal alterations such as translocations, but the precise chromosomal bands and breakpoints involved in the identified translocations may be inaccurate due to the very low resolution of this technique.

Here we report on a small blue round cell tumor with an unusual combination of histological and immunohistochemical findings. Results from standard first line molecular cytogenetic studies turned out to be misleading for both diagnosis and therapy. A complete workup including karyotype analysis, multicolor FISH and construction of new FISH probes was required for the definitive diagnosis of what we consider to represent a variant of clear cell sarcoma bearing an *EWSR1-CREB1 *fusion transcript and expressing an aberrant immunophenotype.

## Case presentation

### Clinical details

54-year-old female presented with pain and swelling of one year duration in her left leg. An MRI scan revealed a 7 cm enhancing mass lying in the posterior calf, at the level of popliteus muscle and extending through the interosseous membrane. The tibial nerve and popliteal vessels were encased in the tumor. Systemic imaging revealed no metastases.

A core needle biopsy was taken, but proved insufficient for a definitive diagnosis. An open biopsy was performed and a working diagnosis of soft tissue Ewing sarcoma rendered. The patient received pre-operative systemic chemotherapy as for Ewing sarcoma, but failed to respond with any tumor shrinkage.

For local treatment of her tumor she was advised to undergo above knee amputation due to the anticipated poor functional results of limb salvage in this situation. Despite extensive counseling and corroborating second opinions she refused amputation. As limb salvage was technically feasible, she underwent pre-operative radiation therapy and a complex wide resection of the tumor was performed, with tibial nerve resection, vascular reconstruction with saphenous vein grafts, allograft reconstruction and internal fixation of the tibial defect, as well as reconstruction of the soft tissue defect with a free tissue transfer from the scapular region. Wide margins were achieved. The patient developed a wound infection with methicillin-resistant *S. aureus *14 days postoperatively. At day 22, she suffered an anastomotic leak of the vessel reconstruction. Although vascularity was restored and her limb at this stage was still viable, she was very disappointed with the functional results of her procedure and requested an above knee amputation. She was clear of disease at last follow up.

## Results

### Core needle biopsy

**Figure 1:**
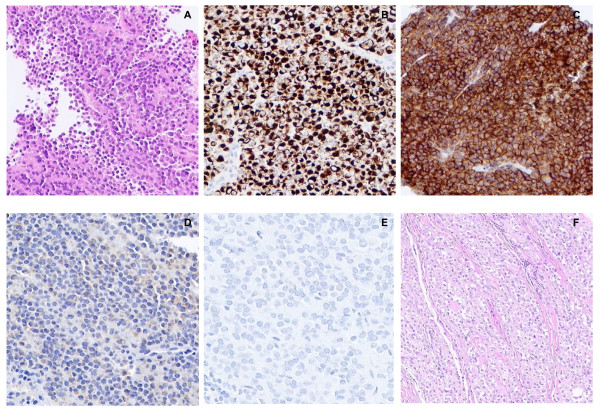
**Microscopic appearance and immunohistochemical features of the tumor**. **A**) Representative area of the core needle biopsy specimen showing a homogeneous plasmacytoid appearance of the tumor cells (H&E; ×100 magnification). **B) **Desmin and **C) **CD99 strong immunoreactivity (×200 magnification). **D) **S100 and **E) **HMB-45 immunohistochemical staining (×200 magnification). **F) **Representative area of the resection specimen showing nests of tumor cells with clear cytoplasm divided by thin fibrous septa (×100 magnification).

Sections showed a small blue round cell tumor growing in poorly cohesive sheets. The tumor cells had uniform plasmacytoid cytology (Fig. 1). A morphologically suspected diagnosis of plasma cell myeloma was ruled out based on the lack of immunoreactivity for kappa or lambda light chains. Instead, the tumor cells showed striking CD99, synapthophysin and desmin immunoreactivity. In contrast, other immunohistochemical markers were all negative, including melanocytic markers such as S-100, HMB-45 and Melan-A. The results of immunohistochemistry are shown in Table 1 with some relevant immunohistochemical staining patterns illustrated in Figure 1.

**Table 1:** Methods and results of immunohistochemistry

Antibody	Source	Dilution/antigen retrieval	Detection method	Reactivity
**CK AE1/AE3**	Dako	1:200/protease digestion	SA/Bi	-
**CK7**	Dako	1:200/protease digestion	SA/Bi	-
**CK20**	Dako	1:500/protease digestion	SA/Bi	-
**TTF1**	Dako	1:100/CC1/95°C/30 min	SA/Bi	-
**EMA**	Dako	1:200/CC1/95°C/30 min	SA/Bi	-
**Vimentin**	Biogenex	1:10000/CC1/95°C/30 min	SA/Bi	-
**CD20**	Dako	1:250/CC1/95°C/30 min	SA/Bi	-
**CD30**	Dako	1:50/CC1/95°C/30 min	SA/Bi	-
**CD34**	Cell Marque	1:50/CC1/95°C/30 min	SA/Bi	-
**Kappa light chain**	Dako	1:5000/protease digestion	SA/Bi	-
**Lambda light chain**	Dako	1:10000/protease digestion	SA/Bi	-
**CD68**	Dako	1:800/CC1/95°C/8 min	SA/Bi	-
**S-100**	Univ. of Toronto	1:1000/CC1/95°C/30 min	SA/Bi	- *
**HMB-45**	Dako	1:100/untreated	SA/Bi	-
**Melan-A**	Cell Marque	1:50/CC1/95°C/30 min	SA/Bi	- *
**PNL2**	Private source	RTU/untreated	Polymer based	-
**SMA**	Dako	1:200/untreated	SA/Bi	-
**Desmin**	Dako	1:200/CC1/95°C/30 min	SA/Bi	+++
**H-Caldesmon**	Dako	1:200/CC1/95°C/30 min	SA/Bi	-
**Myogenin**	Dako	1:50/CC1/95°C/60 min	Polymer based	-
**Myo-D1**	Dako	1:60/citrate buffer (pH6.0)	Polymer based	-
**CD99**	Signet	1:20/CC1/95°C/30 min	SA/Bi	+++
**Synaptophysin**	Cell Marque	1:250/CC1/95°C/30 min	SA/Bi	+++
**Fli1**	BD Pharmingen	1:20/CC1/95°C/60 min	Polymer based	-
**WT1**	Dako	1:50/CC1/95°C/60 min	Polymer based	-
**Ki 67**	Lab Vision	1:200/CC1/95°C/30 min	SA/Bi	>10%

Dako, Carpinteria, CA, USA; Biogenex, San Ramon, CA, USA; Cell Marque, Rocklin, CA, USA; Signet, Dedham, MA, USA; BD Pharmingen, Franklin Lakes, NJ, USA.

SA/Bi: Streptavidin/biotin method; RTU: ready to use; CC1: Cell conditioning 1 (pH8.0) (Ventana medical systems, Tucson, AZ, USA).

*Negative on the core and open biopsies, but patchy positive staining was identified in the resection specimen.

The main differential diagnoses were considered to be Ewing family tumor and alveolar rhabdomyosarcoma. Alveolar rhabdomyosarcoma was ruled out by negative myogenin and myoD1 and by absence of *PAX3/PAX7-FKHR *translocations by FISH. *EWSR1 *FISH, in contrast, showed a break-apart signal pattern in most of the interphase nuclei. A provisional diagnosis of Ewing sarcoma was rendered. However, the histology, the absence of Fli1 staining and the strong desmin immunoreactivity were all considered unusual by several local and consultant pathologists who reviewed the case.

Additional FISH assays for *WT1*, *CHN *and *DDIT3 *(3' partners of *EWSR1 *in desmoplastic round cell tumor, extraskeletal myxoid chondrosarcoma and round cell liposarcoma, respectively) were negative. All core needle biopsy tissue had been consumed after these assays.

In view of the major therapeutic implications engendered by a diagnosis of Ewing sarcoma, a decision was made to perform an open biopsy to obtain more tissue including fresh tissue for cytogenetics.

### Open biopsy

An open biopsy was performed 6 weeks after the original core needle procedure. Histological examination of this specimen revealed plasmacytoid cells with small, eccentric, uniform, moderately vesicular nuclei, most with a single central nucleolus. The tumor cells were arranged in poorly cohesive sheets with no associated necrosis and occasional mitotic figures were evident. Results of immunohistochemical studies were similar to those found on the core biopsy. Other immunohistochemical markers were ordered and found to be negative, including EMA, CD68, PNL2 and WT1.

**Figure 2:**
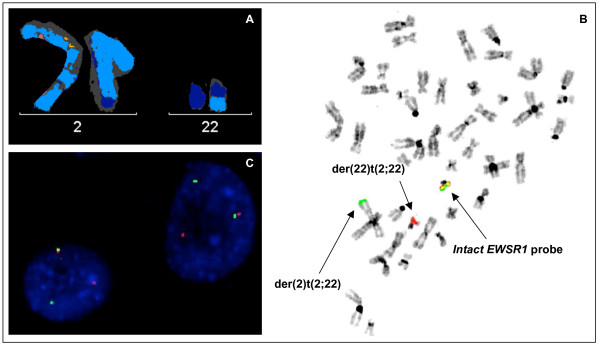
**Characterization of the t(2;22) by molecular cytogenetic techniques**. **A) **Partial multicolor karyotype showing normal chromosome 2, normal chromosome 22 and both derivative chromosomes resulting from t(2;22)(q?36;q?12). **B) **Metaphase FISH with *EWSR1 *break-apart probe. An intact dual-color *EWSR1 *signal can be seen on normal chromosome 22, a green signal on the der(2) and a red signal on the der(22). **C) **Interphase FISH with the *CREB1 *break-apart probe showing break-apart signals (1 fused, 1 green and 1 red signals).

The cultured tumor specimen did not yield metaphases of sufficient quality to be analyzed by G-banding. Multicolor karyotyping identified a few metaphases that contained a reciprocal translocation between 2 q and 22 q in the context of additional numerical and structural changes. The M-FISH stem line karyotype was established as 46, XX, t(2;22)(q?36;q?12), +5, t(14;21)(q?;q?), -21 [5] (Fig. 2). On this basis the diagnosis of Ewing sarcoma was considered confirmed as these findings were consistent with the variant t(2;22) *EWS-FEV *fusion. At this point neoadjuvant chemotherapy was initiated for Ewing sarcoma.

### Characterization of the translocation t(2;22)

To confirm the suspected *EWS-FEV *translocation, a dual-color break-apart probe for *FEV *on chromosome band 2q36 [6] was applied to the cultured specimen. The scoring with this probe revealed two intact signals in the interphase nuclei, with one metaphase showing an intact copy of *FEV *abnormally located on der(22)t(2;22). FISH for *EWSR1 *was performed on the same slide and showed rearrangement in most of the interphase nuclei with the 3' probe signal located on der(2)t(2;22) (Fig. 2b). These findings indicated that *FEV *was not rearranged as expected, and that the breakpoint on the der(2)t(2;22) was in fact centromeric to the *FEV *locus.

A candidate gene approach was initiated to establish the variant *EWSR1 *fusion partner. *CREB1 *is the only other gene in this region previously identified in sarcoma translocations, and a break-apart *CREB1 *probe was created and applied to the cultured specimen. With this FISH experiment, break-apart signals in ~80% of the interphase nuclei were observed (Fig. 2c). The reciprocal t(2;22)(q34;q12) was confirmed using a *EWSR1-CREB1 *dual-fusion probe that revealed the expected fusion signals.

**Figure 3:**
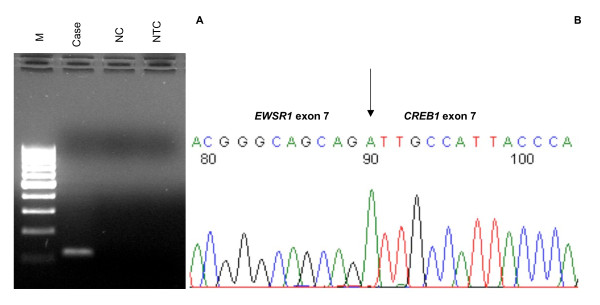
**Agarose gel of *EWSR1-CREB1 *RT-PCR product and sequence of the junction point**. **A) **RT-PCR product of ~120 bp corresponding to the predicted size of the fragment spanning the break point based on the pair of primers used. M, 100 bp molecular marker; NC, negative control (molecularly confirmed clear cell sarcoma with an *EWSR1-ATF1 *translocation); NTC, no template control. **B) **Sequence electropherogram showing an *EWSR1 *exon7/*CREB1 *exon 7 in-frame fusion.

Agarose gel electrophoresis of the RT-PCR product showed a ~120 bp band (Fig. 3a). The size of the product corresponded to the predicted product size of the fragment spanning the fusion transcript breakpoint based on the primer design from published cDNA sequences for *EWSR1 *(NM_013986.2) and *CREB1 *(NM_004379.3). Cloning and sequencing of this product showed an in-frame fusion between *EWSR1 *exon 7 and *CREB1 *exon 7 (Fig. 3b).

Following this workup and considering also the lack of response to neoadjuvant therapy, a diagnosis of clear cell sarcoma with unusual histology and variant immunophenotype was considered.

### Surgical specimen

The resection specimen revealed a high-grade sarcoma with areas similar to those found in the biopsies, but also other areas with neoplastic cells with clear cytoplasm arranged in a nested pattern (Fig. 1f). Extensive infiltration of malignant cells through soft tissues and the dense fascia of the periosteum and interosseous membrane was evident as was extensive lymphovascular invasion. Immunohistochemistry was repeated and while HMB45 remained negative and CD99 strongly positive, S-100 and Melan-A now revealed patchy positive staining. These new findings led us to favor a final diagnosis of clear cell sarcoma.

## Discussion

Clear cell sarcoma (CCS) is an aggressive neoplasm of uncertain histogenesis, accounting for 1% of soft tissue sarcomas. The deep soft tissues of the distal extremities are most frequently involved, often in association with tendons and aponeuroses [7]. Unlike most sarcomas, CCS has a high propensity for lymph nodes metastasis.

The tumor cells show immunoreactivity for melanocytic markers [8,9] as they contain melanosomes in different stages of development [10], and display melanocytic gene expression signatures [11]. Despite its similarity with melanoma, CCS is a distinct entity genetically characterized by the presence of a chromosomal translocation involving *EWSR1 *most frequently partnered with *ATF1 *[10,12-14]. More recently, the alternative chimeric transcript *EWSR1-CREB1 *has been described in three cases of clear cell sarcoma of the gastrointestinal tract that, interestingly, did not show overt melanocytic differentiation [15]. The same chimeric transcript, resulting from a presumed t(2;22)(q34;q12) has been described in three cases of CCS of soft tissue to date [13,14]. Hisaoka *et al *[13] found this chimeric transcript in 2 of 33 cases of CCS (6%). One presented as a 1.5 cm mass in the finger of a 67-year-old male, and the other as a 15 cm mass in the pelvis of a 31-year-old female. Wang *et al *[14] found the *EWSR1-CREB1 *chimeric transcript in 1 of 15 cases (~7%), a superficial tumor in the palm of a 66-year-old female.

*EWSR1-ATF1 *and *EWSR1-CREB1 *are not exclusively found in CCS. They also are the most common gene fusions in angiomatoid fibrous histiocytoma, a mesenchymal neoplasm of borderline malignancy of children and young adults [16,17] that typically presents as a small superficial mass with a distinctive histology (nodules of histiocytoid cells, pseudoangiomatoid blood-filled spaces, fibrous pseudocapsule with a pericapsular lymphoplasmacytic infiltrate). None of these typical clinical features nor morphologic hallmarks were present in the current tumor.

Typically, CCS is composed of nests of plump spindle cells with clear to pale eosinophilic cytoplasm, separated by delicate fibrous septa. The histology of the present tumor was misleading as it presented as a monotonous small round cell neoplasm, with most cells exhibiting plasmacytoid characteristics. Deviation from the usual histology has been seen in some cases of CCS [10,13,18]. Areas with rhabdoid tumor cells were detected in 24% and 16% of the molecularly confirmed cases reported by Antonescu *et al *[10] and Hisaoka *et al *[13]. However, to the best of our knowledge, desmin expression by CCS cells has not been described previously [13,19] and CD99 immunoreactivity has been found only in one molecularly confirmed case of CCS of the stomach [20]. Nevertheless, these are markers that are not consistently tested in the clinical workup for clear cell sarcoma, as small blue round cell tumors such as Ewing sarcoma and rhabdomyosarcoma are not part of the usual histological differential diagnosis of CCS.

CCS of soft tissue is typically characterized by the expression of S-100 and melanosome-associated markers [10,13,14,18,21]. In this regard too, the present tumor had a unique immunoprofile, as S-100, HMB45 and Melan-A were all negative in both the core needle and incisional biopsies. It was only in the resection specimen that some patchy staining for S-100 and Melan-A was seen. Lack of vimentin expression was also an unexpected finding.

In a setting of supportive histomorphology and immunohistochemistry, detection of *EWSR1 *rearrangements by FISH is a very useful diagnostic tool that can support the diagnosis for the known set of *EWSR1*-translocation bearing tumors (Ewing family tumors, clear cell sarcoma, extraskeletal myxoid chondrosarcoma, desmoplastic round cell tumor, and variant myxoid/round cell liposarcomas) [4]. Nevertheless, in light of the lack of specificity of *EWSR1 *break apart probes in the differential diagnosis of small round cell sarcomas, the search for a 3' partner is very important in the follow-up analysis when the histology is not entirely classic for one of these diagnoses. The combined genome wide-screen provided by multicolor FISH, and the advantage of metaphase FISH to detect rearrangements with reference to specific chromosomal bands allowed us to interrogate the involvement of *CREB1 *on chromosome band 2q34 as the *EWSR1 *3' partner in the reciprocal t(2;22).

The rate of detection of diagnostic chimeric transcripts in CCS by RT-PCR ranges between 91%-100% among published studies [10,12-14,18]. This minor difference could be explained by the fact that not all the studies were intended to cover all the transcript variants, and the negative cases may well represent tumors with overlooked alternative chimeric transcripts. Interestingly, the rate of detection of *EWSR1 *rearrangement in CCS by FISH has been variously reported to be 70%, 88% or 100% [18,22,23], raising the possibility of *EWSR1 *being substituted by other genes as a 5'partner in a small subset of CCS.

Our case would represent the fourth soft tissue CCS reported harboring this fusion transcript variant, and the first in which the cytogenetic features of this reciprocal translocation t(2;22)(q34;q12) are detailed. The predicted structure of EWSR1-CREB1 in this case is similar to that described previously [14-16] in which the oncogenic chimeric transcript retains its CREB1 carboxyl-terminal basic leucine zipper DNA binding and dimerization domain, fused to the amino-terminal transcriptional activation domain of EWS which confers oncogenic properties by transcriptional dysregulation.

## Conclusions

This case supports the fact that *EWSR1-CREB1 *is not a translocation variant exclusive to clear cell sarcomas arising in the gastrointestinal tract.

The described variation in histology and immunohistochemical features displayed by CCS must be taken into account when considering the differential diagnosis for an unusual small blue round cell tumor. In this setting, immunohistochemistry and even karyotype can be misleading, and FISH for both of the translocation partners or PCR primers accounting for all of the fusion transcript variants is important for accurate diagnosis.

## Methods

### Tissue handling

The core needle biopsy was fixed in neutral-buffered formalin and processed for routine histology. Unstained 6 μm paraffin sections were submitted for interphase FISH. Representative tissue from the incisional biopsy and from the excision specimen were submitted fresh for cytogenetic analysis and a portion was snap frozen for molecular studies. The remaining tissue was fixed and submitted for routine histology and immunohistochemistry.

### Immunohistochemistry

Standard immunohistochemical studies were done using a Ventana Benchmark XT Instrument (Ventana medical systems, Tucson, AZ, USA). The source and dilution of the antibodies, antigen retrieval and the detection methods are presented in Table 1.

### Cytogenetic studies, FISH and multicolor FISH

Chromosome analysis was performed by standard methods after 6 days culture in RPMI 1640 medium supplemented with 20% fetal calf serum and L-glutamine. Metaphase chromosomes were banded by the GTG method and the karyotypes were described according to the International System for Human Cytogenetic Nomenclature 2005 [24].

FISH was performed on sections from the core needle biopsy and on cell preparations from the cultured incisional biopsy specimen. Commercial probes for *EWSR1*, *FUS *and *DDIT3 *(Abbott Molecular, Des Plaines, IL, USA) and "in-house" dual-color break-apart (*CHN*, *WT1*, *FEV*) and dual-color dual-fusion (*PAX3-FKHR *and *PAX7-FKHR*) bacterial artificial chromosome (BAC) probes were used. An "in house" dual-color break-apart probe was prepared for the detection of *CREB1 *rearrangements using BACs RP11-354H1 and RP11-135B21. An "in-house" dual-color, dual-fusion *EWSR1-CREB1 *probe was prepared to confirm the reciprocal t(2;22)(q34;q12) using BACs RP11-135B21/RP11-354H1 (chromosome 2) and RP11-945M21/RP11-1126O13 (chromosome 22).

The BAC probes were directly labeled by nick translation using either Spectrum Green or Spectrum Orange (Abbott Laboratories, Abbott Park, IL, USA). The chromosomal locations of the BACs were initially confirmed by hybridization to normal metaphases from a peripheral blood culture. Each probe was scored by counting 200 interphase nuclei under fluorescent microscopy. For confirmation of true breakapart, >10% of cells showing a clear pattern of one fused, one red and one green signal was required. For confirmation of dual fusions, >5% of nuclei with a clear two fused, one red, one green pattern was required.

Multicolor FISH was performed using the 24 XCyte color kit (Metasystems, Altlussheim, Germany) following the manufacturer's protocol.

### Reverse transcription-PCR of the fusion transcript

Total RNA from the frozen open biopsy tumor tissue was isolated using the RNeasy mini kit (Qiagen, Maryland, USA). Two micrograms of total RNA were reverse transcribed using the qScript cDNA SuperMix system (Quanta Biosciences, Maryland, USA) and used as template for PCR amplification of the *EWS-CREB1 *fusion breakpoints using the following primers [14] EWSex7-F1 primer (5'-TCCTACAGCCAAGCTCCAAGTC -3') and CREB1ex7-REVC primer specific for CREB1 (5'-GTACCCCATCGGTACCATTGT -3'). The PCR amplification started with 5 minutes at 95°C; followed by 35 cycles of 30 seconds at 95°C, 30 seconds at 58°C, and 45 seconds at 72°C; and a final extension of 10 minutes at 72°C. PCR product was detected by 1.5% agarose gel electrophoresis.

### cDNA cloning and sequencing analysis of the fusion transcript

The cDNA of the break-point-crossing fragment of the *EWS-CREB1 *chimeric transcript was first amplified with HindIII-EWSex7-FW (5'TATCaagcttTCCTACAGCCAAGCTCCAAGTC) and XhoI-CREB1ex7-RV (5'- TTTTctcgagGTACCCCATCGGTACCATTGT), then subcloned into pcDNA3.1 (+) vector. Purified plasmid DNA was verified using the same restriction enzymes. The clones with an insert of the appropriate size were then submitted for cDNA sequencing using a T7-FW primer.

## Consent

Written informed consent was obtained from the patient for publication of this case report. A copy of the written consent is available for review by the Editor-in-Chief of this journal.

## Competing interests

The authors declare that they have no competing interests.

## Authors' contributions

MP carried out the candidate gene approach and the preparation of the "in-house" FISH probes, led the molecular studies, performed the cloning of the fusion transcript and drafted the manuscript. DEH led the cytogenetic and molecular cytogenetic studies, their analysis and interpretation and reviewed the manuscript. MMH was involved in the histopathologic diagnosis of the patient's sample and the review of the manuscript. PWC provided the clinical data of the patient and helped drafting the manuscript. HH organized the original histopathologic diagnosis of the patient. TON initiated and led the pathologic investigations and co-wrote the manuscript. All authors read and approved the final manuscript.
